# Design and Fabrication of Customised Diabetic Insoles for Optimised Foot Pressure Distribution Using Finite Element Analysis and Additive Manufacturing Technology

**DOI:** 10.3390/bioengineering12111217

**Published:** 2025-11-07

**Authors:** Jafar Mala, Hossein Bisheh

**Affiliations:** Department of Mechanical Engineering, School of Engineering and Built Environment, Anglia Ruskin University, Chelmsford, Essex CM1 1SQ, England, UK; jafarmalamn5@gmail.com

**Keywords:** diabetic foot, insole, finite element analysis, optimisation, 3D printing

## Abstract

Diabetic foot ulcers (DFUs) are a serious complication of diabetes and a leading cause of lower-limb amputations. Excessive plantar pressure in high-risk regions such as the heel and forefoot contributes significantly to their development. This study presents the design, simulation, and three-dimensional prototyping of customised diabetic insoles aimed at redistributing pressure and reducing ulcer risk. Insole models are created in Autodesk Inventor, evaluated with finite element analysis (FEA) in ANSYS Workbench, and fabricated using 3D printing technology. Three designs are evaluated, i.e., a standard insole, a circular-cutout insole, and an irregular-cutout insole, using four different materials, i.e., Ethylene Vinyl Acetate (EVA)1, EVA2, EVA3, and polyurethane (PU). Under applied pressure of 0.3 MPa by a diabetic foot, a customised EVA1 insole reduces barefoot peak stress from 3.97 MPa to 1.51 MPa (61.96% reduction), while irregular isolations lower ulcer-rim stress from 0.87 MPa to 0.63 MPa (27.8% reduction). EVA-based insoles outperformed the PU one, and prototypes are successfully printed in EVA and PU, demonstrating feasibility for low-cost and patient-specific applications.

## 1. Introduction

**Figure 1:**
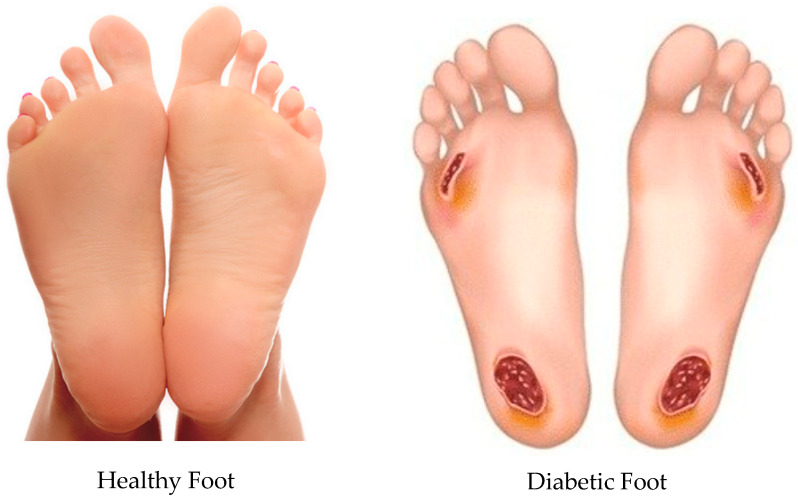
Comparison of a normal foot versus a diabetic foot. (Reprinted with permission from Refs. [4,5]).

Diabetes mellitus is a chronic metabolic condition of rising global prevalence, profoundly impacting both public health and economic stability. As of 2021, over 537 million adults worldwide were living with diabetes, a figure projected to rise to 643 million by 2030, underscoring the growing healthcare challenge it presents [1]. Among its most severe and costly complications are diabetic foot ulcers (DFUs) as shown in Figure 1. These wounds primarily result from three main factors: nerve damage (peripheral neuropathy) that reduces protective sensation, impaired blood circulation (peripheral artery disease) causing poor tissue oxygenation, and altered foot mechanics that lead to concentrated pressure points on the foot’s bottom surface [2]. The diabetic foot has scar tissue buildup in the areas shown in Figure 1. This means that these areas will have much higher stresses due to scar tissue and swelling that is a continual cycle that leads to skin breakdown. DFUs frequently lead to chronic and non-healing wounds, significantly increasing the risk of infection, hospitalisation, and non-traumatic lower-limb amputations, which remain a devastating outcome for patients. Beyond the profound physical suffering, DFUs drastically diminish patients’ quality of life, contribute to chronic pain, and impose immense psychological distress. Furthermore, the management of DFUs places a substantial economic burden on healthcare systems globally, with annual expenditures frequently reaching billions of dollars [3]. Consequently, there is an urgent and critical need for effective, accessible, and scalable interventions that can precisely manage plantar pressures to prevent and treat DFUs. 

For the prevention and management of diabetic foot ulcers, customised orthotic insoles are broadly accepted as a key non-invasive therapeutic approach. These meticulously engineered medical devices are designed to precisely redistribute plantar pressure away from anatomical regions particularly susceptible to breakdown, such as the metatarsal heads, heels, and other high-pressure bony areas, thereby mitigating the excessive mechanical stresses that cause tissue damage. Unlike common and mass-produced options, customised insoles clearly provide better pressure relief and are precisely fitted to each patient’s unique foot shape and movement requirements [6]. However, the widespread adoption and accessibility of customised insoles are significantly limited by their traditional manufacturing processes. These conventional methods often involve labour-intensive techniques such as plaster casting, manual adjustments, and multiple iterative rounds of physical prototyping and fitting. Such processes are time-consuming, expensive, and require specialised expertise, which collectively limit their availability, especially in low-resource settings where the burden of DFUs is often high [7]. The slow and expensive nature of traditional insole production shows a critical global need for faster, cheaper, more sustainable, and more scalable ways to design and make personalised diabetic foot orthoses.

In recent years, the field of medical device design has undergone a profound transformation driven by advancements in computational modelling, particularly FEA which has been used as an exceptionally powerful numerical technique for simulating complex biomechanical interactions within the human body [8,9,10,11,12,13,14], offering a robust and versatile platform for the virtual testing and optimisation of orthotic designs. Through FEA, engineers can accurately predict how an insole will perform under various realistic loading conditions, such as standing or dynamic phases of walking (e.g., heel strike, mid-stance, and toe-off), by precisely modelling the intricate interplay between the foot, the insole, and the applied external forces. This sophisticated capability not only significantly accelerates the design iteration cycle but also provides invaluable quantitative and qualitative insights into localised pressure distribution, stress concentrations, and strain patterns across the plantar surface. Such detailed insights are crucial for making informed design modifications aimed at optimising patient-specific pressure relief by eliminating the necessity for iterative physical prototypes during the preliminary design and optimisation phases. FEA substantially streamlines the development process and drastically reduces both development costs and material waste, thereby promoting more environmentally sustainable manufacturing practices [15].

Over past decades, numerous research studies have been conducted on the efficacy of diabetic insoles in foot pressure relief [6,16,17,18,19,20,21,22,23,24,25,26,27,28,29,30,31]. Paton et al. [17] designed custom-made functional insoles to reduce risk factors for ulceration of neuropathic diabetic feet in comparison with prefabricated insoles. Their custom-made functional insole was slightly more effective than the prefabricated insole in reducing the forefoot pressure time integral at issue (27% vs. 22%). However, they found that the custom-made insoles are more expensive than prefabricated insoles evaluated in their study, with no better output in peak pressure reduction. A considerable and growing body of research has successfully utilised FEA to investigate insole efficacy and the biomechanical responses of the diabetic foot. For instance, Chanda and Unnikrishnan [23] conducted a pivotal study employing FEA to evaluate a novel design of insoles featuring specific ulcer isolations for post-ulcer diabetic foot conditions. Their simulations, which included a full-scale foot model with ulcers of varying geometries and sizes, demonstrated substantial peak stress reductions (up to 91.5%) at ulcer sites, particularly for irregular ulcer geometries, and identified critical material stiffness thresholds for optimal offloading. While this work provided foundational insights into ulcer offloading through computational means, it carried certain limitations. Their foot model, for example, simplified the complex anatomical structures by representing the foot as a single linear elastic entity, rather than differentiating between individual bones, ligaments, and soft tissues, which could impact the accuracy of highly localised stress distributions [23]. Furthermore, their analysis did not explicitly incorporate the effects of footwear, which can substantially influence plantar pressure distribution, and explored a restricted range of ulcer geometries (circular and some irregular shapes). Mahesh and Ramachandran [24] developed a finite element (FE) modelling to evaluate the performance of functionally graded elastomers for the application of diabetic footwear. Through 3D FEA using ANSYS, a uniaxial compression test was performed on five different densities of PU foam and respective stress–strain curves were obtained and the distribution of stresses versus time due to weight of the person were observed for further optimisation. In a study by Tang et al. [25], FEA was used to design customised insoles and optimise the stress distribution of the contact surfaces between the foot and the insole by considering functional gradient structural properties of the insole. They fabricated the customised insoles using additive manufacturing technology and tested them under mechanical loading where the results showed that the customised design of insoles increases the foot contact area by approximately 30% and decreases the peak contact pressure by 35%. Niu et al. [29] proposed a novel custom layered modular insole with eight layers of small cushions for a diabetic foot and used FEA to make a comparison between the traditional insoles and the proposed one. The results of their research work showed that the proposed custom insole reduces the peak pressure from 208.86 to 160.02 kPa with observation of no high pressure in the sensitive locations of the diabetic foot, although it increases the insole volume and squeezes the space available for the foot.

Although significant studies have been dedicated to the use of FEA in optimising various aspects of insole design, including material properties and specific design features, for effective pressure relief, they often still operate within a hybrid design paradigm, combining computational simulation with subsequent physical prototyping for validation. Additionally, many existing computational analyses rely on generic foot models or simplified load conditions, potentially limiting the true personalisation achievable for highly individualised biomechanical responses or complex, irregular ulcer morphologies encountered in clinical practice.

Thus, a critical research gap persists in the current landscape, despite the proven efficacy of custom insoles and the power of FEA, the widespread translation of highly effective custom insole designs into accessible clinical practice remains significantly hindered by development processes that even when incorporating simulations, still largely rely on costly, time-consuming, and material-intensive physical prototyping for optimisation and validation. There is a pressing and unmet need for a purely simulation-based design and validation framework that is sufficiently comprehensive to accurately account for diverse patient-specific anatomical variations and complex ulcer geometries, thereby eliminating the need for iterative physical prototypes during the entire design and optimisation phase.

This paper directly addresses these critical limitations by presenting a novel and robust simulation framework for the comprehensive design and computational validation of customised diabetic insoles using advanced FEA. The presented approach rigorously models and optimises various insole designs, systematically exploring the biomechanical efficacy of different material properties (specifically, various densities of EVA and PU) and a wide range of geometric configurations, including those precisely tailored for both circular and highly irregular ulcer relief. By leveraging the advanced predictive power of FEA to precisely minimise peak plantar pressures and effectively offload high-risk areas under realistic loading conditions, this study significantly accelerates the entire design cycle, substantially reduces development costs, and minimises the material waste associated with traditional iterative prototyping. This methodology thereby establishes a highly cost-effective and environmentally sustainable pathway for personalised DFU management. Furthermore, the practical feasibility and manufacturability of these simulation-validated designs are critically demonstrated through their successful physical realisation using accessible Fused Deposition Modelling (FDM) 3D printing technology, offering a viable and scalable pathway for future clinical application.

In this study, through a precise Computer-Aided Design (CAD) modelling by Autodesk Inventor, the foot (with and without ulcers) and customised insole designs (for with and without ulcers) are modelled and then an advanced FEA using ANSYS Workbench 2024 R2 is used to simulate walking conditions on the designed insole models with assigned properties of EVA and PU materials. By presenting the distribution of stress on the foot and insole models, their performance on reducing foot pressures is investigated and discussed for diabetic ulcer conditions. Finally, physical prototypes of designed insoles are fabricated using the FDM technique of additive manufacturing technology.

## 2. Materials and Methods

This section explains the steps and procedures used in the study, from designing the insole and human foot in Autodesk Inventor 2024 CAD software to running FEA simulations in ANSYS Workbench to evaluate stress and pressure distribution. The study primarily follows a simulation-based approach to ensure efficient and cost-effective development; while physical prototypes are later fabricated to demonstrate manufacturability, no physical testing is used for validation. Multiple insole designs are created and assembled with a modelled diabetic foot to assess different conditions, including no ulcers, circular ulcers, and irregular ulcers. The methodology covers CAD modelling, material selection, and finite element simulations, with justifications for design choices to ensure the insole meets the requirements of diabetic foot care. FEA provides a non-invasive approach to study stress distribution and plantar pressure effects, which is crucial for understanding and managing diabetic foot complications [32].

### 2.1. Design and Simulation Procedure

The research approach for this project is entirely simulation-based and focuses on designing, assembling, and assessing customised insoles for diabetic patients using FEA. The process is broken down into key stages allowing for flexibility in comparing different insole designs and foot conditions:

i. CAD Modelling: The insole models are created using Autodesk Inventor based on the UK size 6 dimensions. Different versions are modelled: one with no cutouts, one with circular ulcer relief zones, and one with irregular ulcer cutouts.
ii. Human Foot Modelling: A simplified model of the human foot is developed and matched to the insole dimensions. Three versions are made: a normal foot, one with circular ulcers, and another with irregular ulcers.
iii. Assembly of Foot and Insole: Each foot version is assembled with the corresponding version of the insole model in Autodesk Inventor for further simulation of realistic loading scenarios.
iv. Material Selection: Materials are selected based on biomechanical needs such as shock absorption, pressure relief, and comfort. EVA foams are the main choice due to its frequent use in diabetic footwear. PU has also been selected as the material of the insole.
v. FEA Simulation: Once models are assembled and materials assigned, static structural analysis is conducted in ANSYS Workbench to evaluate stress distribution and overall performance under applied pressure simulating the walking and stance loading conditions.

This research design allows for a structured comparison of how different insole types affect stress levels in the diabetic foot, particularly in areas with and without ulcers.

#### 2.1.1. Insole Design and Material Selection

The insole design process is based on biomechanical principles to ensure optimal support, pressure relief, and stability for diabetic patients. The approach includes CAD modelling, material selection, and design enhancement to improve functionality. Justifications for each choice are made based on literature and industry standards.

#### 2.1.2. CAD Modelling of Insoles

The insole is designed using Autodesk Inventor, with standard foot dimensions based on the size 6 dimensions. This size is selected because it is widely used in orthopaedic research and serves as a practical benchmark in academic and clinical studies related to foot biomechanics and diabetic foot care [32]. The design process follows biomechanical principles to enhance stability, pressure redistribution, and shock absorption.

### 2.2. CAD Modelling Workflow

The CAD modelling phase plays a critical role in replicating the biomechanics of a diabetic foot and the performance of various insole configurations. The designs are developed using Autodesk Inventor and follow a structured workflow to ensure anatomical accuracy, pressure relief, and comparative analysis across different foot and insole conditions.

**Figure 2:**
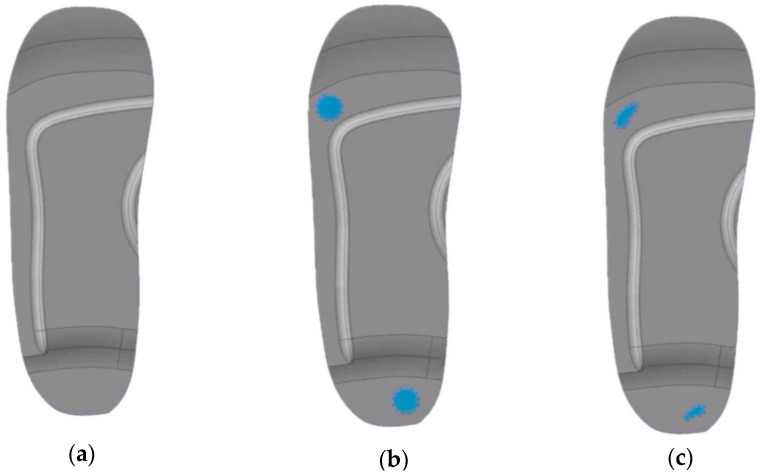
Customised insole designs: (**a**) no ulceration, (**b**) circular ulceration, and (**c**) irregular ulceration.

**Figure 3:**
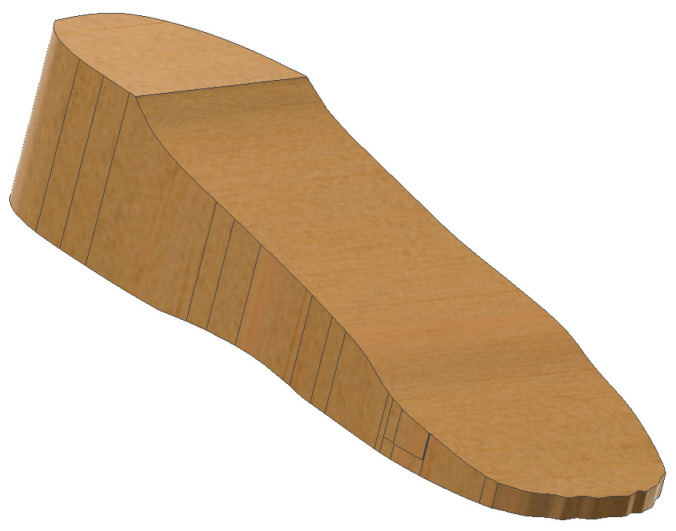
Human foot model design.

**Figure 4:**
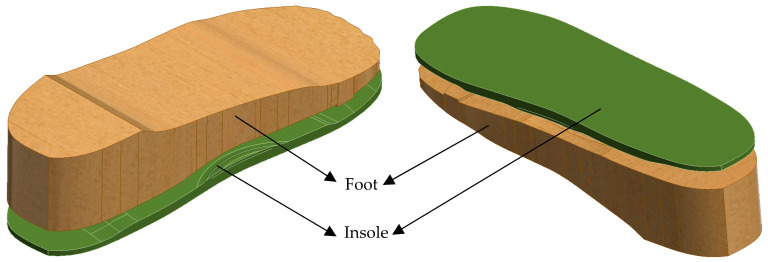
Assembly of a human foot on a customised insole.

To ensure an anatomically accurate and functional diabetic insole, the CAD model is developed in the following stages:

i. Initial Insole Design Without Ulcer Cutouts
  - A base insole is created using UK size 6 dimensions.
  - The insole is extruded to a 5 mm thickness.
  - Functional features like arch support (12 mm height) and heel cushioning (3 mm recess) are incorporated.
  - A heel cup is also added to stabilise the rearfoot when bearing weight (see Figure 2a).
ii. Insole Design with Circular Ulcer Cutouts
  - Based on medical literature and pressure mapping zones, two circular cutouts are made: one on the heel region and another on the ball of the foot (metatarsal area) as shown in Figure 2b.
  - These cutouts are introduced to offload pressure from the ulcer zones during standing.
  - The design is saved as a separate file to facilitate direct comparison with other models.
iii. Insole Design with Irregular Ulcer Cutouts
  - A more complex version of the insole is modelled by introducing irregularly shaped cutouts on the heel and forefoot areas as shown in Figure 2c.
  - These are based on common ulcer geometries observed in diabetic patients and are manually shaped using spline profiles.
  - This model is saved independently to evaluate the pressure redistribution effectiveness of more tailored insole designs.
iv. Foot Model Development
  - A simplified human foot is modelled to simulate pressure application (see Figure 3).
  - Three variations in the foot are designed: normal foot (no ulcers), diabetic foot with circular ulcers (heel and ball), and diabetic foot with irregular ulcers (heel and ball).
  - The foot model is sized to anatomically match the UK size 6 insole.
v. CAD Assembly
  - Each version of the foot is assembled with the corresponding insole in Autodesk Inventor as shown in Figure 4.
  - These assemblies allow proper alignment for realistic boundary conditions and load application during FE simulations.
  - These systematic design stages enable a controlled comparison of insole performance in ulcer management and foot pressure mitigation under diabetic conditions.

### 2.3. Materials Selection

FEA simulations using ANSYS Workbench are conducted for four various materials, i.e., EVA1 (Shore A65), EVA2 (Shore A85), EVA3 (Shore A95), and PU. Each material is assessed under three different foot conditions: a normal diabetic foot with no ulcers, a diabetic foot with circular ulcers, and a diabetic foot with irregular ulcers. This allows us to assess how material stiffness and compliance influence plantar pressure distribution and stress concentration under various pathological scenarios. However, EVA foam is recommended more for its lightweight nature, compressibility, and excellent energy absorption properties. It minimises peak plantar pressures, making it ideal for diabetic patients [33]. Research studies also show that EVA foam improves long-term comfort and reduces overall foot stress [34]. Table 1 lists properties of materials used in this study.

In addition to the insole materials selection, a customised material is also defined for the human foot in ANSYS Workbench to simulate diabetic tissue properties. Based on the study of Ref. [23], the foot is modelled as a linear elastic material with a modulus of elasticity of 483 MPa and a Poisson’s ratio of 0.4 (see Table 2). These values represent an averaged property across the plantar soft tissue to simplify the simulation. A new material is added into the *Engineering Data* section of ANSYS Workbench, and the material properties of the human foot are manually entered to assign them to the modelled foot.

### 2.4. Finite Element Analysis

**Figure 5:**
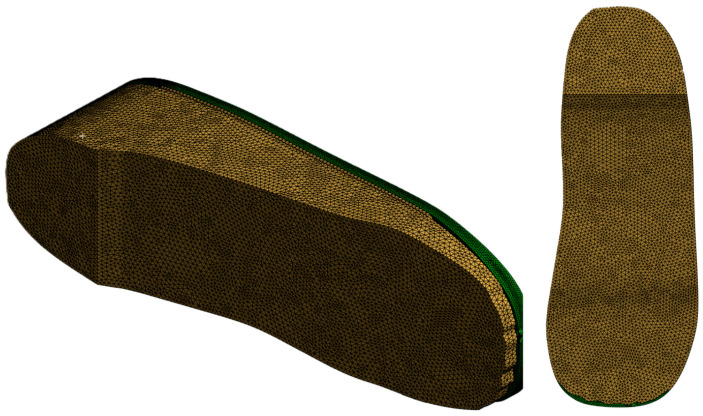
Meshing process: a meshed human foot on the custom insole model.

**Figure 6:**
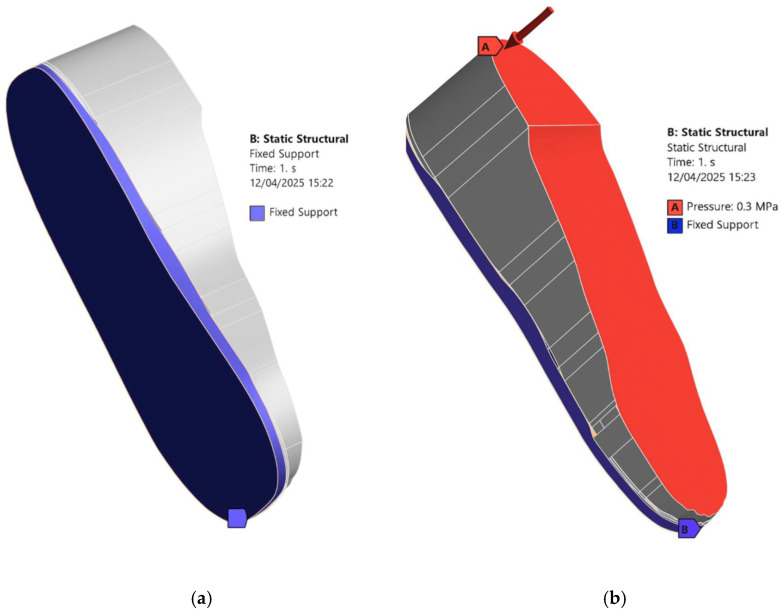
Applied loading and boundary conditions: (**a**) the fixed support selection, and (**b**) the pressure application.

To evaluate the mechanical performance of the insole and its effect on plantar pressure distribution, ANSYS Workbench is used to conduct FEA. The steps below outline the entire simulation workflow followed for each condition and material setup:

i. Importing the Geometry: The assembled CAD models (insole and foot) are exported as a STEP file from Autodesk Inventor and imported into the *Static Structural* module in ANSYS Workbench.
ii. Assigning Material Properties: EVA1, EVA2, EVA3, and PU material properties, as listed in Table 1, are selected from the ANSYS library and applied to the imported insole models. In the *Engineering Data* section, a new material is defined with the linear elastic material properties listed in Table 2 to assign foot material properties to the imported foot model.
iii. Mesh Generation: The entire assembled insole and foot model is meshed with quadratic element type and appropriate mesh size is determined through a convergence study. Extra care is taken to ensure finer meshing around ulcer zones and contact areas to improve simulation accuracy (see Figure 5).
iv. Loading and Boundary Conditions: In the *Static Structural* module, a fixed support is applied to the bottom of the insole and side edges that would be in contact with the ground or shoe interior [see Figure 6a]. A pressure load is applied to the top surface of the foot as shown in Figure 6b. The magnitude of the applied pressure is set to 0.1 MPa, 0.2 MPa, and 0.3 MPa to represent different stance loading conditions. These values are chosen based on the magnitudes considered in the study of Ref. [23] to simulate plantar pressures applied to the diabetic feet under static loading conditions. Selecting this range allows us to stay within physiologically relevant loading scenarios and observe how the insole material and design influence the stress distribution. Figure 6 describes the applied loading and boundary conditions.
v. Solution Setup: In the solution setup, the output parameter is Equivalent von Mises stress, which is used to evaluate stress distribution in the foot and insole. The simulation is then executed in ANSYS Workbench.

Unless otherwise stated, all simulations and reported percentage changes are at a uniform load of 0.3 MPa to ensure internal consistency across comparisons. This process is repeated for all insole materials and foot conditions (no ulcer, circular ulcers, and irregular ulcers) to collect and compare results across different scenarios.

## 3. Results and Discussion

This section presents the results obtained from the FEA of the diabetic foot rested on the customised insoles designed using Autodesk Inventor and evaluated in ANSYS Workbench. The simulations are conducted to assess the insole’s effectiveness in redistributing plantar pressure and reducing stress concentrations on the diabetic foot under various conditions.

The results include validation study against the literature, mesh convergence study, and comparisons between barefoot and insole-supported scenarios, as well as with and without ulcer isolations. Different material configurations (EVA1, EVA2, EVA3, and PU) are examined to observe how material stiffness affects the stress distribution and deformation. The performance of the insoles is analysed for both circular and irregular ulcer scenarios to determine their therapeutic effectiveness. Additionally, the feasibility of fabricating the customised insoles using the additive manufacturing technology is discussed, highlighting the potential for low-cost and patient-specific solutions in diabetic foot care.

### 3.1. Validation

To ensure the accuracy and credibility of the FEA, the results of this study are compared with the results of Ref. [23] as summarised in Table 3. Ref. [23] analysed plantar stresses under stance loads ranging from 0.1 to 0.3 MPa but reported their headline results at 0.3 MPa. In their study, barefoot peak stress at 0.3 MPa was approximately 0.23 MPa, which was reduced to about 0.17 MPa with a customised insole. In comparison, the present study under pressure of 0.3 MPa (Cohort A) observed barefoot peak stress of 3.97 MPa when standing on the hard ground and a reduction in stress to 1.51 MPa when the foot is standing on EVA1 customised insole, corresponding to a 61.96% decrease. Although the absolute magnitudes differ due to modelling assumptions such as simplified linear elastic foot, single-material configuration, and simplified boundary conditions, the qualitative trends remain constant with the results of Ref. [23]. Both studies demonstrate that compliant insoles substantially reduce peak stresses and ulcer isolations offload stress from high-pressure regions, particularly in irregular ulcers. This consistency supports the biomechanical plausibility of the CAD-based insole design and the FEA used in this study.

### 3.2. Mesh Convergence Study

Before proceeding with the main simulations, a mesh convergence study is conducted to ensure the accuracy and reliability of the results. Therefore, it is important to strike a balance by identifying a mesh size where further refinement has minimal impact on the results; this is termed mesh convergence.

To perform this analysis, the effect of different finite element mesh sizes, ranging from 1 mm to 10 mm, are assessed for the von Mises stress for a diabetic foot standing on a customised insole made of EVA1 for the non-ulcerated condition, as well as for a customised insole without ulcer isolations made of EVA1. For each case study, simulations are conducted at three applied pressures, namely 0.1 MPa, 0.2 MPa, and 0.3 MPa, to observe also the effect of load variations on the evolution of peak von Mises stress toward convergence.

**Figure 7:**
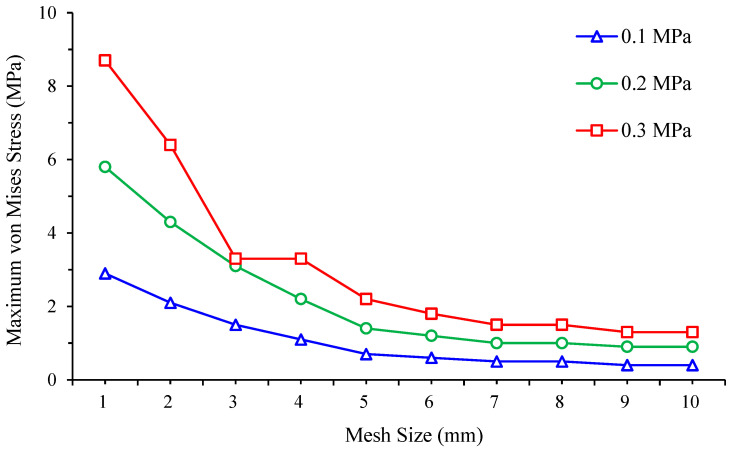
Mesh convergence study: von Mises stress generated on the foot without ulcers standing on a customised insole made of EVA1 under applied pressures of 0.1, 0.2, and 0.3 MPa for various finite element mesh sizes.

**Figure 8:**
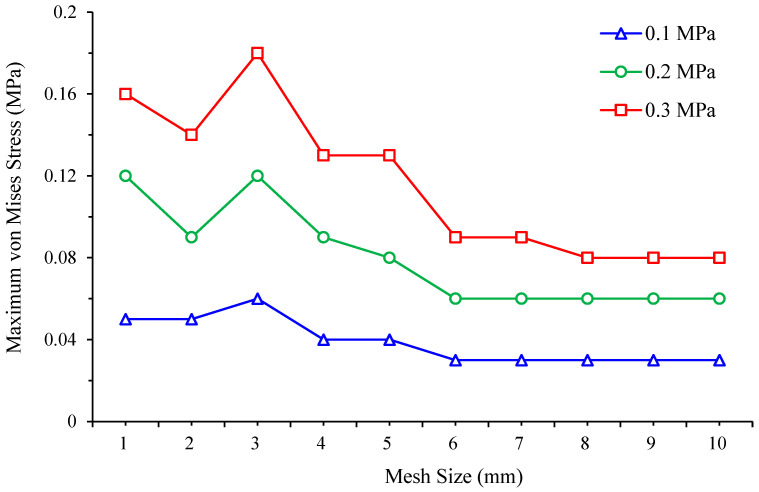
Mesh convergence study: von Mises stress in a customised insole made of EVA1 under applied pressures of 0.1, 0.2, and 0.3 MPa whilst standing for various finite element mesh sizes.

Table 4 and Figure 7 present the foot peak von Mises stress values and trends under three different foot pressures obtained for various mesh sizes. Convergence is judged using the foot response at 0.3 MPa pressure because plantar foot stress is the primary outcome in this study. Between the two finest meshes, the foot peak stress changed from 1.50 MPa at 7 mm mesh size to 1.52 MPa at 8 mm mesh size, a relative difference of 1.3%, which is below the 2% convergence tolerance. As listed in Table 5 and plotted in Figure 8, the insole response shows the same trend and is reported for completeness but is not used to set the mesh. On this basis, a finite element mesh size of 7 mm is selected and set for all simulation runs using ANSYS Workbench. Unless otherwise stated in the following results, all comparisons to Ref. [23] and all ulcer analyses are reported at 0.3 MPa (Cohort A) pressure.

### 3.3. Diabetic Foot Stresses in a Barefoot Without Ulcer Conditions and with Custom Insoles

**Figure 9:**
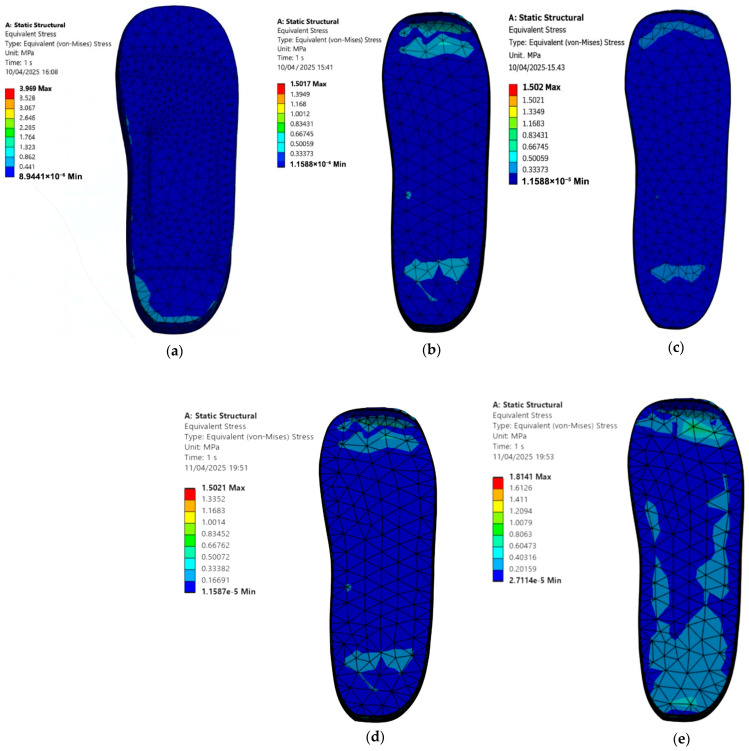
Von Mises stress distribution for a non-ulcerated foot standing on (**a**) hard and flat ground and on customised insoles made of (**b**) EVA1, (**c**) EVA2, (**d**) EVA3, and (**e**) PU under applied pressure of 0.3 MPa (Cohort A).

**Figure 10:**
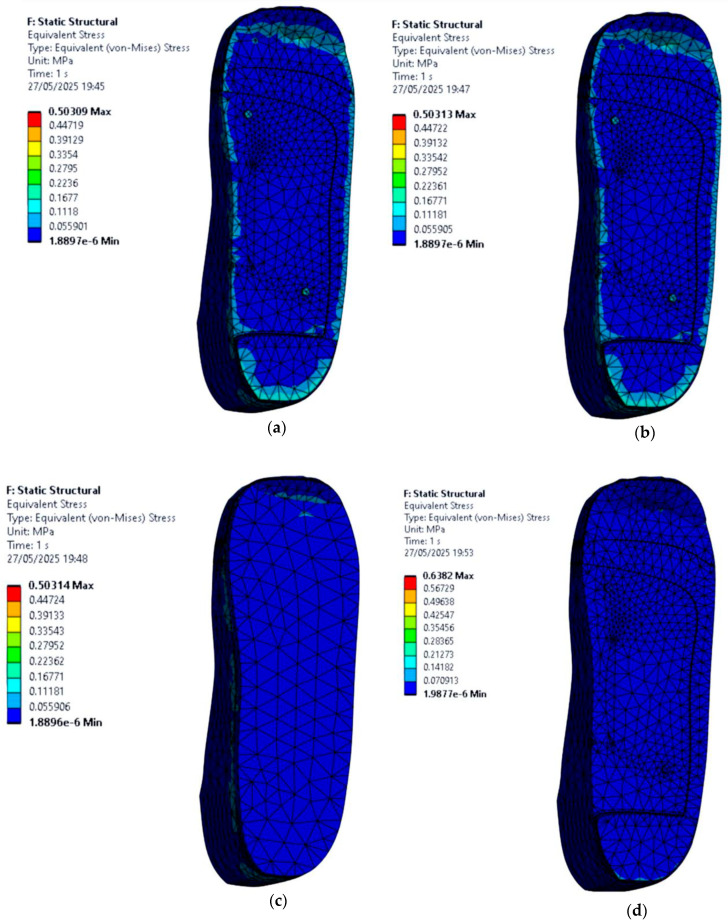
Von Mises stress distribution for a non-ulcerated foot standing on customised insoles made of (**a**) EVA1, (**b**) EVA2, (**c**) EVA3, and (**d**) PU under applied pressure of 0.1 MPa (Cohort B).

To establish a baseline, a standing simulation is conducted for a diabetic foot without ulcers placed directly on a hard and flat ground surface, using the material properties listed in Table 1 and Table 2. Uniform pressures of 0.3 MPa in Cohort A and 0.1 MPa in Cohort B are applied to replicate stance loading configuration. Figure 9 shows the barefoot case and the foot standing on customised insoles under 0.3 MPa pressure, while Figure 10 shows the corresponding cases under 0.1 MPa pressure. The setup, constraints, and applied pressure remained consistent within each cohort.

The simulation results demonstrate that all customised insoles reduced plantar stresses on a diabetic foot compared with a barefoot standing on a hard and flat ground. The peak von Mises stress values on the foot are:

Cohort A, 0.3 MPa:
  - Barefoot: 3.97 MPa.
  - EVA1: 1.50 MPa.
  - EVA2: 1.50 MPa.
  - EVA3: 1.50 MPa.
  - PU: 1.81 MPa.
Cohort B, 0.1 MPa:
  - EVA1: 0.50 MPa.
  - EVA2: 0.50 MPa.
  - EVA3: 0.50 MPa.
  - PU: 0.64 MPa.

Both cohorts show the same qualitative outcome. Customised insoles substantially reduce plantar stresses on a diabetic foot relative to a barefoot standing on hard and flat ground. EVA-based insoles, particularly EVA1 and EVA2, consistently produce lower peak stresses than the PU one at their respective load levels. These findings confirm that even without ulcer-specific isolations, selecting appropriate insole materials can significantly reduce plantar loading and stress and may help in preventing ulcer formation.

### 3.4. Stress Distribution on the Foot with Circular Ulcers Standing on Customised Insoles (Cohort A, 0.3 MPa)

This section focuses on evaluating how circular ulcers affect stress distribution in a diabetic foot under stance loading configuration, and how a customised insole design can help offload pressure in these vulnerable areas. Two circular ulcers are modelled, one at the heel and one at the ball of the foot, since these regions typically experience the highest plantar pressures in diabetic patients.

Simulations are conducted under an applied pressure of 0.3 MPa using four insole materials: EVA1, EVA2, EVA3, and PU. For each material, two scenarios are considered: one where the foot with circular ulcers stands on a standard insole without ulcer relief zones, and another where the same circularly ulcerated foot stands on a customised insole featuring circular cutouts beneath the ulcer sites.

**Figure 11:**
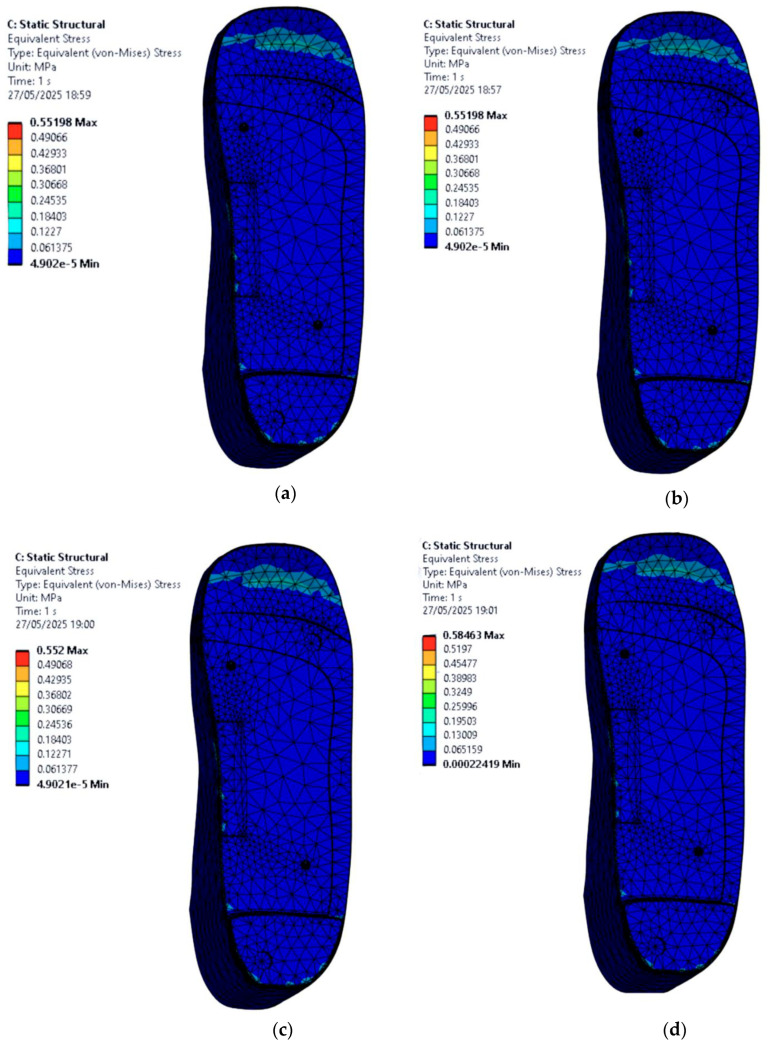
Von Mises stress distribution for a circular-ulcerated foot standing on customised insoles without ulcer isolations made of (**a**) EVA1, (**b**) EVA2, (**c**) EVA3, and (**d**) PU under an applied pressure of 0.3 MPa (Cohort A).

From the FE results shown in Figure 11, stress is significantly higher in the ulcer zones when no isolation cutouts are included in the insole. The peak von Mises stress values on the circular-ulcerated foot for each material case of the insole are as follows:

- EVA1: 0.55 MPa.
- EVA2: 0.55 MPa.
- EVA3: 0.55 MPa.
- PU: 0.58 MPa.

In this model, the PU-based insole produces the highest local peak stress at the heel ulcer compared with the EVA-based insoles. Among the EVA materials, EVA1 and EVA2 offered better overall performance.

**Figure 12:**
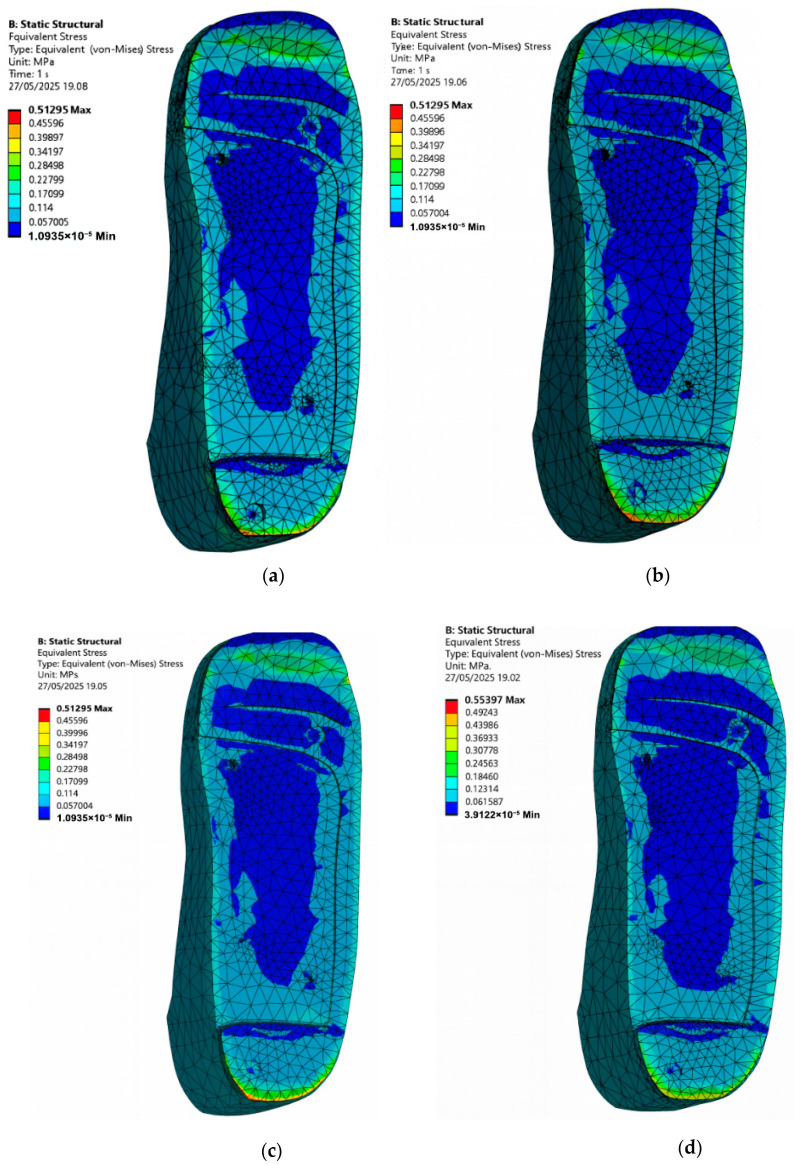
Von Mises stress distribution for a circular-ulcerated foot standing on customised insoles with circular isolations made of (**a**) EVA1, (**b**) EVA2, (**c**) EVA3, and (**d**) PU under an applied pressure of 0.3 MPa (Cohort A). The ulcer-rim peak is lower with the circular isolation.

However, when ulcer isolation cutouts are introduced in the insole design, there is a noticeable reduction in the foot peak stress under the affected areas across all four materials as shown in Figure 12. These relief zones effectively redistribute the load to healthier surrounding tissue, which is essential in preventing further ulcer deterioration or delayed healing. The new peak von Mises stress values on the circular-ulcerated foot for each material case of the insole are as follows:

- EVA1: 0.51 MPa.
- EVA2: 0.51 MPa.
- EVA3: 0.51 MPa.
- PU: 0.55 MPa.

It is observed that at 0.3 MPa pressure, the circular isolation reduces ulcer-rim peak stress by 7.27% for EVA1–EVA3 (0.55 to 0.51 MPa) and by 5.17% for PU (0.58 to 0.55 MPa). EVA1 and EVA2 again stood out, showing lower peak stresses while maintaining structural integrity. Overall, this part of the study demonstrates that material selection alone is not sufficient; geometric modifications such as ulcer isolations also play a key role in reducing plantar stress. The combination of EVA-based materials and targeted offloading designs appears to be the most effective strategy for addressing circular ulcers in diabetic foot conditions.

### 3.5. Stress Distribution on the Foot with Irregular Ulcers Standing on Customised Insoles (Cohort A, 0.3 MPa)

**Figure 13:**
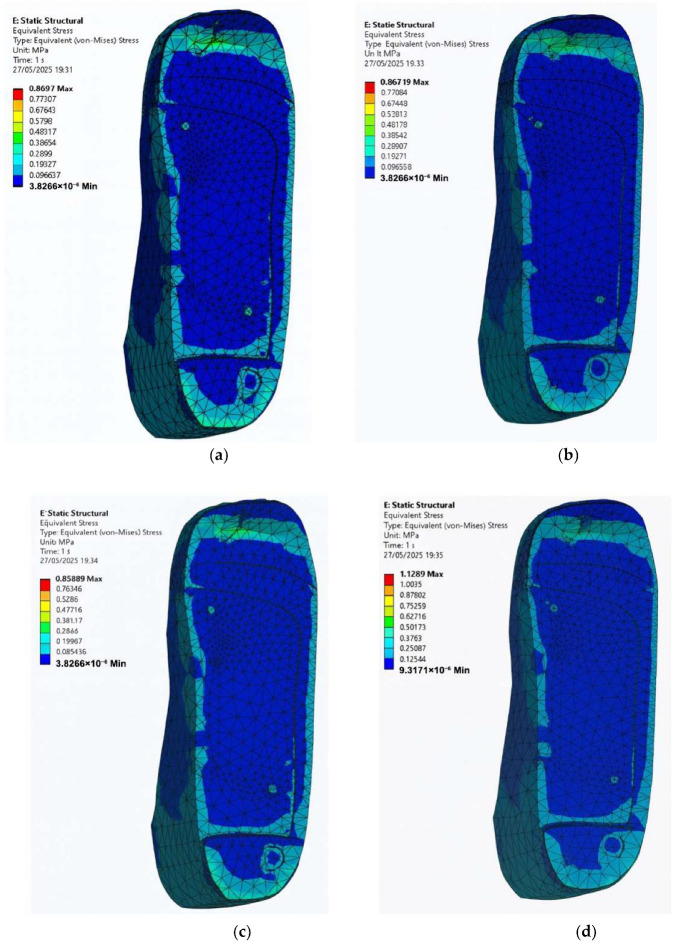
Von Mises stress distribution for an irregular-ulcerated foot standing on customised insoles without ulcer isolations made of (**a**) EVA1, (**b**) EVA2, (**c**) EVA3, and (**d**) PU under an applied pressure of 0.3 MPa (Cohort A).

In many real-life diabetic cases, foot ulcers do not always present in neat and circular shapes. Instead, they tend to appear irregular and uneven across pressure-prone areas like the heel and forefoot as shown in Figure 1. To study how these irregular ulcer shapes affect stress distribution during the standing configuration, a diabetic foot model is simulated with irregular ulcers at the heel and forefoot, standing on customised insoles made of EVA1, EVA2, EVA3, and PU under an applied pressure of 0.3 MPa. For the first set of simulations, no ulcer relief or isolation zones are included in the insole. These results are shown in Figure 13 for the four materials with following peak von Mises stress on the irregular-ulcerated foot:

- EVA1: 0.87 MPa.
- EVA2: 0.87 MPa.
- EVA3: 0.86 MPa.
- PU: 1.13 MPa.

As seen in the plots of Figure 13, the ulcer-rim peaks are highest in ulcer-prone regions, especially at the heel, indicating greater risk of discomfort and tissue damage. This reinforces the importance of insole modifications specifically tailored to offload these areas.

**Figure 14:**
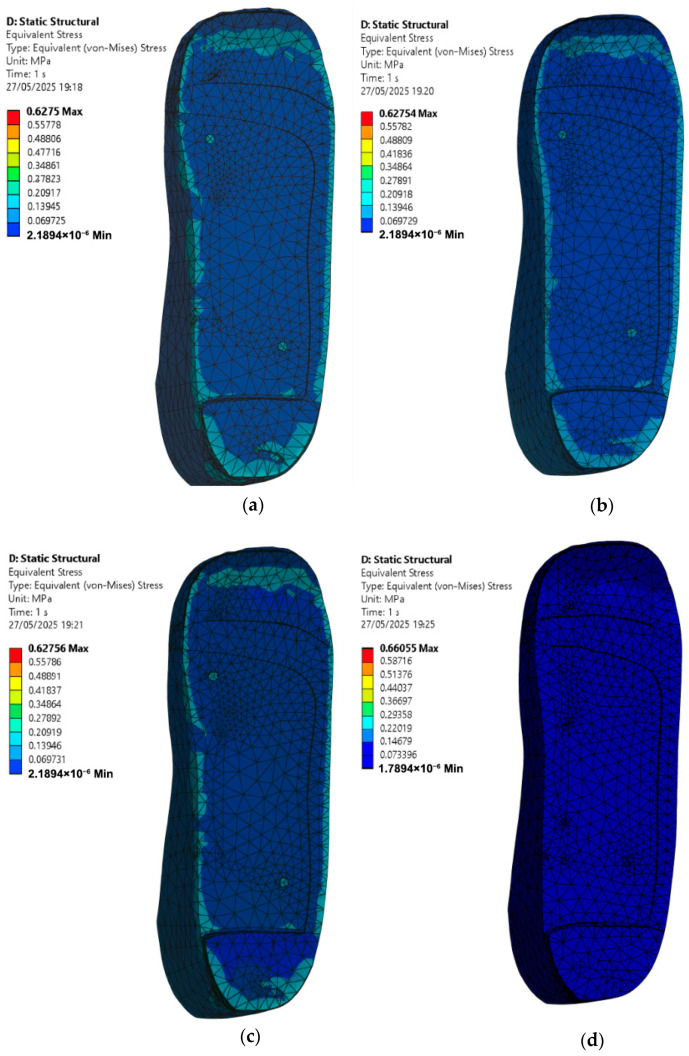
Von Mises stress distribution for an irregular-ulcerated foot standing on customised insoles with irregular isolations made of (**a**) EVA1, (**b**) EVA2, (**c**) EVA3, and (**d**) PU under an applied pressure of 0.3 MPa (Cohort A). The ulcer-rim peak is lower with irregular isolation.

To assess the impact of irregular ulcer isolations, the insole CAD models are modified to include irregular cutouts matching the ulcer regions on the diabetic foot. A second set of simulations is then run under the same boundary conditions and 0.3 MPa applied pressure. The updated results are shown in Figure 14 for EVA1, EVA2, EVA3, and PU. The new peak von Mises stress values on the irregular-ulcerated foot are as follows:

- EVA1: 0.63 MPa.
- EVA2: 0.63 MPa.
- EVA3: 0.63 MPa.
- PU: 0.66 MPa.

All four materials show clear reductions when irregular isolations are present, with the largest reduction observed for PU in this model. These results highlight that geometry modifications by considering ulcer-specific isolations in customised insoles can substantially reduce localised stress concentrations and may support ulcer healing or prevention. The qualitative trend agrees with the reference study [23], and all interpretations are made within the 0.3 MPa cohort.

### 3.6. Effect of Insole Material Properties on Foot Stress

**Figure 15:**
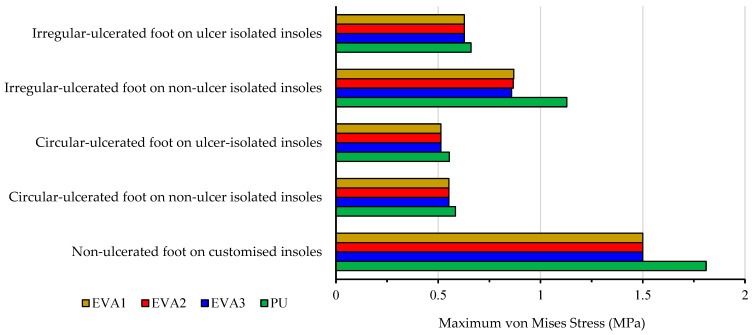
Effect of insole materials and ulcer conditions on the foot peak von Mises stress at 0.3 MPa pressure (Cohort A).

To evaluate how material properties influence peak stress on the foot, the maximum von Mises stresses are compared for five different foot conditions using four customised insole materials, i.e., EVA1, EVA2, EVA3, and PU. The five scenarios include a normal diabetic foot (no ulcers), a foot with circular ulcers (with and without ulcer isolations on the insole), and a foot with irregular ulcers (with and without ulcer isolations on the insole). All simulations are conducted under a standing pressure of 0.3 MPa, and the values are summarised in Table 6 and plotted in Figure 15.

From the results, EVA1 consistently shows the lowest foot peak stress values, particularly for the irregular ulcer condition, which has the highest stress levels due to its non-uniform shape. EVA2 and EVA3 followed closely behind with very minor differences, while PU consistently produced the highest foot stress values in all cases, indicating it may not be the most suitable choice for high-risk diabetic feet.

For circular ulcers, adding isolations did not significantly reduce foot stresses by using the EVA-based insoles, likely due to their already effective pressure-absorbing nature. The PU-based insole showed a slight reduction of the foot stress with isolations, although its performance still lagged EVA-based insoles.

Overall, the results confirm that material stiffness and elasticity significantly affect the stress distribution. Softer EVA variants, particularly EVA1, proved more effective for ulcer prevention and pressure offloading in diabetic foot care. These findings are supported by the contour plots provided in Figure 10, Figure 11, Figure 12, Figure 13 and Figure 14.

## 4. Customised Insole Prototyping Using Additive Manufacturing Technology

To support the FEA results and demonstrate the real-world feasibility, the final insole designs are fabricated using 3D printing at the additive manufacturing laboratory of Anglia Ruskin University. Additive manufacturing technologies have been widely used in design and fabrication of biomedical prostheses and implants [35,36,37,38,39]. It was also reported that using a new class of quasi-zero stiffness (QZS) mechanical metamaterials featuring bio-inspired, variable-thickness curvilinear architectures and sustainable, 3D-printable reinforced bio-composites can be beneficial for 3D printing of orthopaedic prostheses and devices [40].

Hence, the additive manufacturing approach is chosen in this study for its suitability in low-volume and customised prototyping, especially for biomedical products such as diabetic insoles where personalised fit and geometry are crucial.

**Figure 16:**
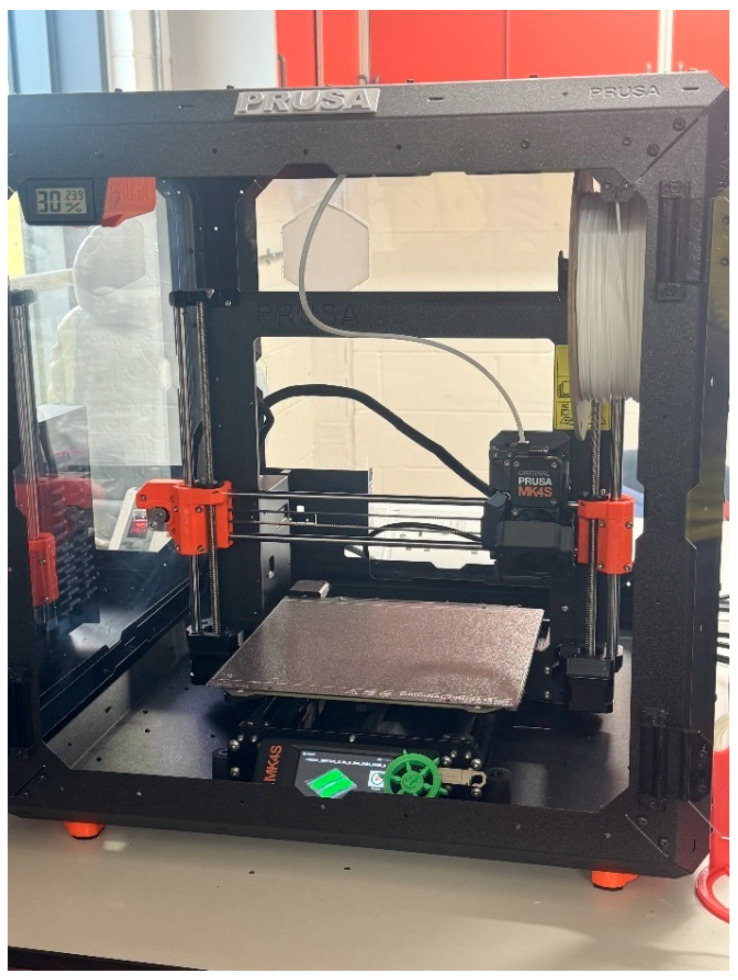
A PRUSA MK4S 3D printer.

The CAD models are exported from Autodesk Inventor as STL files, sliced using PrusaSlicer, and printed on the PRUSA MK4S 3D printer, shown in Figure 16, with the following technical specifications:

- Technology: Fused Filament Fabrication (FFF) or Fused Deposition Modelling (FDM).
- Material: flexible EVA and PU (FLEX/117) filaments.
- Layer height: 0.2 mm.
- Print time: approximately 8 h per specimen.

**Figure 17:**
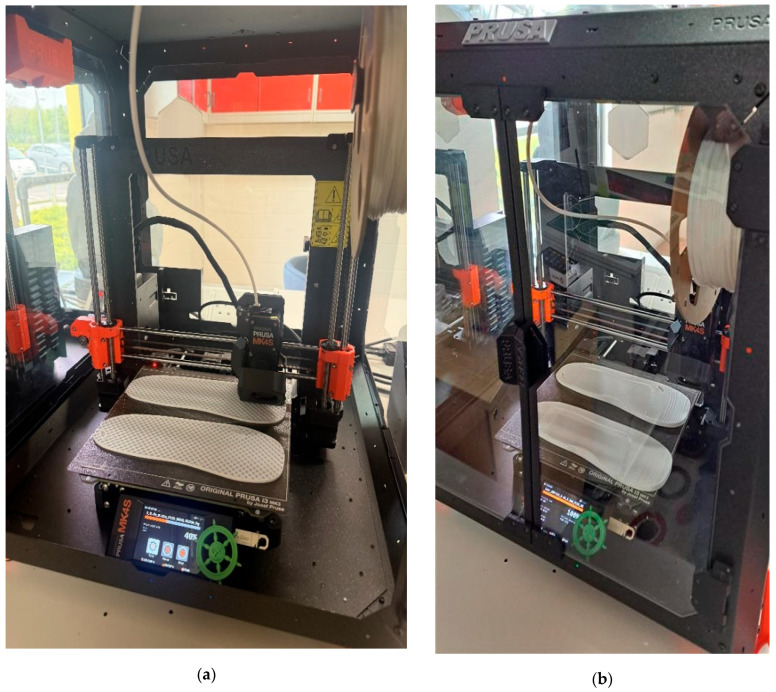
Three-dimensional printing of insole models after (**a**) 3 h and (**b**) 8 h (completed model).

**Figure 18:**
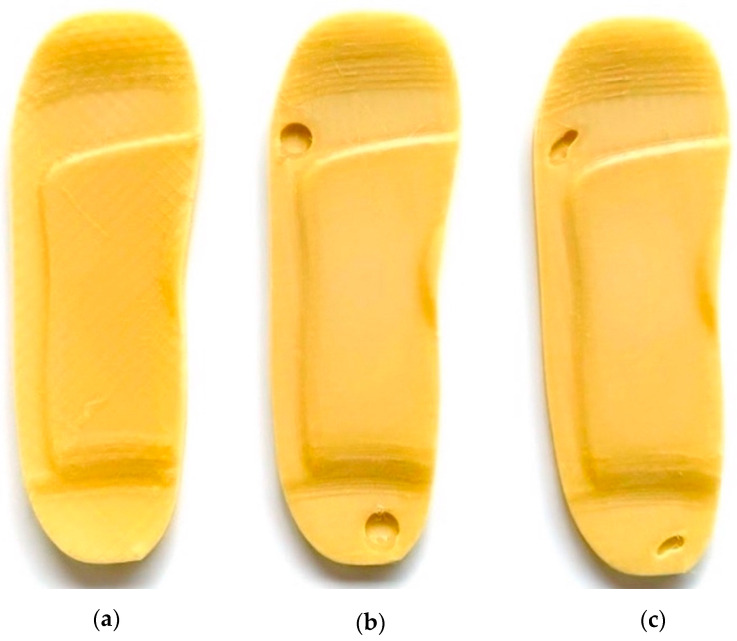
Three-dimensional printed insole specimens made of EVA filament showing (**a**) no ulceration, (**b**) circular ulceration, and (**c**) irregular ulceration.

**Figure 19:**
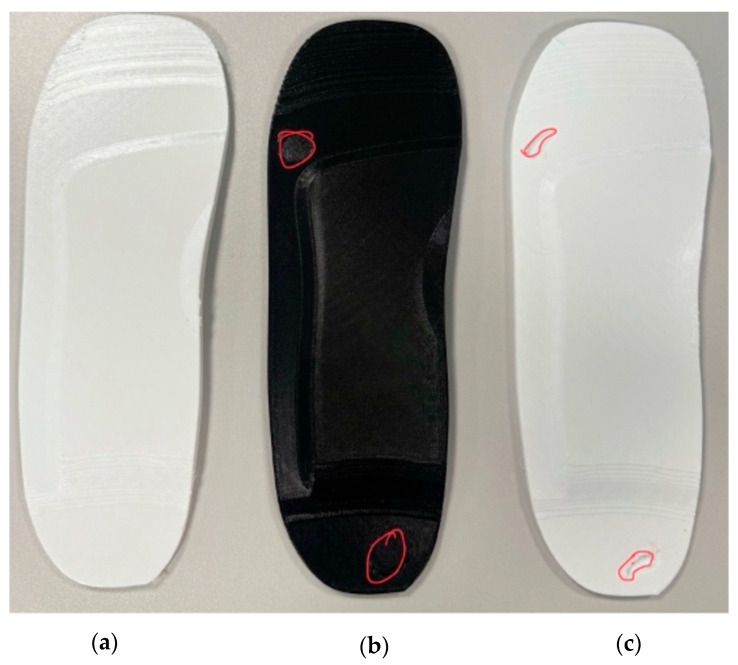
Three-dimensional printed insole specimens made of PU filament showing (**a**) no ulceration, (**b**) circular ulceration, and (**c**) irregular ulceration.

The 3D printing progress after 3 h and 8 h are shown in Figure 17 and finally, three separate insole specimens made of EVA and PU materials are printed:

1. Standard insole—No ulcer cutouts, used as a baseline [Figure 18a and Figure 19a].
2. Circular ulcer relief insole—Two circular cutouts, at the heel and forefoot [Figure 18b and Figure 19b].
3. Irregular ulcer relief insole—Complex cavities shaped according to pressure zones [Figure 18c and Figure 19c].

The EVA and PU filaments have been selected for their good printability and flexibility, which made them suitable for demonstrating pressure offloading zones and anatomical shaping. Key design features such as the arch support, heel cup, and ulcer relief zones are successfully printed with good surface detail and dimensional accuracy in both EVA and PU.

While the printed insoles are not intended for clinical wear, the prototypes confirm that the design can be translated from digital simulation to physical form. This validates the CAD-FEA workflow and provides a foundation for future functional testing and material optimisation.

## 5. Conclusions and Perspective

This research paper set out to design, simulate, and evaluate customised diabetic insoles using a fully digital approach, with the goal of improving plantar pressure distribution for patients at risk of foot ulceration. Through CAD modelling, FEA, and 3D printing, the study delivered a complete development cycle that proved both technically effective and clinically meaningful. All design choices were guided by biomechanical evidence and validated through comparison with academic literature. Key findings and conclusions of this research study are as follows:

- Customised Insole Geometry and Plantar Stress Reduction: When the diabetic foot was simulated in a barefoot condition on a hard and flat ground surface, the peak von Mises stress reached 3.97 MPa. When supported by a customised insole made of EVA1, the stress dropped to 1.51 MPa, representing a 61.96% reduction. This confirms that using insoles can meaningfully offload pressure in high-risk regions such as the heel and forefoot, which is critical for ulcer prevention.
- Ulcer Isolation Cutouts and Local Pressure Relief: For feet with irregular ulcers, adding cutouts at the heel and forefoot regions offloaded stress around the ulcer zones. The peak stress reduced from 0.87 MPa to 0.63 MPa, a 27.58% reduction. While not drastic, this validates the principle that small geometric modifications can improve clinical outcomes, especially when paired with compliant materials.
- Material Selection and Stress Reduction: Among all tested materials, EVA1 (Shore A65) consistently produced the lowest stress values. For example, in the non-ulcer foot condition, EVA1 (1.50 MPa) reduced stress by 17.12% compared to PU (1.81 MPa), which showed the highest values. This demonstrates that softer and more elastic materials are better at absorbing shock and redistributing pressure in diabetic feet.
- Three-Dimensional Printing for Effective and Accessible Prototyping: All three insole models, including the standard design, the circular-cutout design, and the irregular-cutout design, were successfully printed using flexible EVA and PU filaments with the FDM additive manufacturing technique. Each specimen required approximately 8 h of print time, and the process was carried out entirely at the additive manufacturing laboratory of Anglia Ruskin University. This demonstrates that simulation-led workflows can be practically and affordably translated into physical prototypes using standard additive manufacturing technologies.

Based on the findings and challenges encountered during this study, the following recommendations are proposed for future research and product development:

- Advanced Foot Modelling with Hyper-Elastic Tissue: This study used a simplified linear elastic foot material. Future work should implement hyper-elastic models (e.g., Ogden or Mooney–Rivlin) to better capture the non-linear behaviour of plantar soft tissue under compression.
- Patient-Specific Foot Geometry: The current design used a generic UK size 6 foot. Using 3D scans of actual patient feet would allow for customised insole shapes and ulcer relief zones, improving performance and clinical relevance.
- Dual-Material Design Concepts: While this study focused on a single-material approach, performance could be enhanced through composite or dual-material 3D printing, where soft materials (like EVA) are used for comfort zones and firmer materials (like PU or nylon) provide arch support and stability.
- Alternative Prototyping Techniques Beyond FDM: While the FDM technique was practical and effective, other manufacturing techniques may provide improved accuracy and durability:
  ✓ Stereolithography (SLA) allows for high-detail prototypes with better surface finish.
  ✓ Selective Laser Sintering (SLS) can process flexible or composite materials.
  ✓ Injection Moulding is ideal for large-scale production with consistent material properties, although less suited for rapid prototyping due to tooling costs.

Combining rapid 3D printing with traditional moulding techniques could be useful for developing low-cost but durable insoles.

- Sensor Feedback for Physical Validation: Future studies should integrate thin-film pressure sensors into insole prototypes to validate simulation results with actual pressure measurements through physical testing under dynamic loading such as walking trials.
- Optimisation of Ulcer Isolation Shapes: Only one size and shape of cutout was assessed. Future research should experiment with different depths, contours, and geometries, potentially using AI or machine learning to automate patient-specific optimisation.

This research achieved its main aim of demonstrating how simulation-based design, supported by additive manufacturing technology, can be used to develop functional and cost-effective insoles for diabetic patients. The methodology was straightforward, the results aligned with existing research, and the workflow can be replicated in both academic and low-resource clinical settings.

The outcomes highlight real potential for personalising diabetic foot care, especially in contexts with limited access to advanced orthotic services. With further work on material modelling and patient-specific geometry, this approach can evolve into a valuable tool in diabetic foot management.

## Figures and Tables

**Table 1:** Properties of materials used for the insoles (derived from ANSYS library).

Properties	EVA1 (Shore A65, 33% Vinyl Acetate)	EVA2 (Shore A85, 25% Vinyl Acetate)	EVA3 (Shore A95/D50, 12% Vinyl Acetate)	PU (Polyurethane, Flexible)
Density $\left(kg/m^{3}\right)$	950	950	935	31.94
Young’s modulus (Pa)	$7.593\times 10^{6}$	$2.449\times 10^{7}$	$7.937\times 10^{7}$	48,990
Poisson’s ratio	0.4799	0.4799	0.4799	0.2793
Bulk modulus (Pa)	$6.5813\times 10^{7}$	$2.0307\times 10^{8}$	$6.5813\times 10^{8}$	36,996
Shear modulus (Pa)	$2.6816\times 10^{6}$	$8.2742\times 10^{6}$	$2.6816\times 10^{7}$	19,147
Tensile ultimate strength (Pa)	$9.747\times 10^{6}$	$1.649\times 10^{7}$	$1.849\times 10^{7}$	$1.449\times 10^{5}$
Tensile yield strength (Pa)	$9.747\times 10^{6}$	$1.649\times 10^{7}$	$1.849\times 10^{7}$	4135

**Table 2:** Average linear elastic material properties used to model a diabetic foot condition [23].

Linear Elastic Parameters	Modulus of Elasticity (MPa)	Poisson’s Ratio
Diabetic foot model	483	0.4

**Table 3:** Comparison of key simulation parameters and outcomes at 0.3 MPa pressure between the present study and study of Ref. [23]. Reductions are computed within each study relative to the barefoot case.

Comparison Parameter	Present Study	Study of Ref. [23]
Applied pressure: standing position	0.30 MPa	0.30 MPa
Foot peak stress: barefoot on the hard ground versus on the insole	3.97 MPa → 1.51 MPa (61.96% ↓)	0.23 MPa → 0.17 MPa (26.08% ↓)
Foot peak stress with irregular ulcers: non-isolated insole versus isolated one	0.87 MPa → 0.63 MPa (27.58% ↓)	0.93 MPa → 0.08MPa (91.39% ↓)
Foot tissue model	Linear elastic (E=483MPa, ν=0.4)	Hyper-elastic (Ogden model)
Insole materials used	EVA1, EVA2, EVA3, and PU	Multilayer soft foam
Simulation scope	Static structural + 3D prototyping	Static structural + 3D prototyping

**Table 4:** Mesh convergence study: von Mises stress generated on a diabetic foot without ulcers standing on a customised insole made of EVA1 under applied pressures of 0.1, 0.2, and 0.3 MPa for various finite element mesh sizes.

		Peak von Mises Stress (MPa)		
	Pressure (MPa)	0.1	0.2	0.3
Mesh Size (mm)				
1		2.89	5.78	8.67
2		2.13	4.26	6.39
3		1.54	3.07	3.26
4		1.09	2.18	3.26
5		0.72	1.44	2.16
6		0.60	1.21	1.81
7		0.50	1.01	1.50
8		0.51	1.02	1.52
9		0.45	0.90	1.35
10		0.43	0.87	1.30

**Table 5:** Mesh convergence study: von Mises stress in a customised insole without ulcer isolations made of EVA1 under applied pressures of 0.1, 0.2, and 0.3 MPa for various finite element mesh sizes.

		Peak von Mises Stress (MPa)		
	Pressure (MPa)	0.1	0.2	0.3
Mesh Size (mm)				
1		0.05	0.11	0.16
2		0.05	0.09	0.14
3		0.06	0.12	0.18
4		0.04	0.09	0.13
5		0.04	0.08	0.13
6		0.03	0.06	0.09
7		0.03	0.06	0.09
8		0.03	0.06	0.08
9		0.03	0.06	0.08
10		0.03	0.06	0.10

**Table 6:** Peak foot von Mises stress (MPa) for different foot and insole conditions at 0.3 MPa pressure (Cohort A). Conditions refer to insoles with or without ulcer isolations as indicated.

	Peak von Mises Stress (MPa)			
Foot and Insole Conditions	EVA1	EVA2	EVA3	PU
Non-ulcerated foot on customised insoles	1.50	1.50	1.50	1.81
Circular-ulcerated foot on non-ulcer isolated insoles	0.55	0.55	0.55	0.58
Circular-ulcerated foot on ulcer-isolated insoles	0.51	0.51	0.51	0.55
Irregular- ulcerated foot on non-ulcer isolated insoles	0.87	0.87	0.86	1.13
Irregular-ulcerated foot on ulcer isolated insoles	0.63	0.63	0.63	0.66

## Data Availability

The original contributions presented in the study are included in the article, further inquiries can be directed to the corresponding author.
